# The serum angiotensin-converting enzyme 2 and angiotensin-(1-7) concentrations after optimal therapy for acute decompensated heart failure with reduced ejection fraction

**DOI:** 10.1042/BSR20192701

**Published:** 2020-06-15

**Authors:** Shinji Hisatake, Shunsuke Kiuchi, Takayuki Kabuki, Takashi Oka, Shintaro Dobashi, Takahiro Fujii, Takanori Ikeda

**Affiliations:** Department of Cardiovascular Medicine, Toho University Graduate School of Medicine, Tokyo, Japan

**Keywords:** angiotensin converting enzyme 2, angiotensin-(1-7), acute heart failure, optimal therapy

## Abstract

**Objective:** Elucidation of the role of angiotensin-converting enzyme (ACE) 2 (ACE2)/angiotensin (Ang)-(1-7)/Mas receptor axis in heart failure is necessary. No previous study has reported serial changes in ACE2 and Ang-(1-7) concentrations after optimal therapy (OT) in acute heart failure (AHF) patients. We aimed to investigate serial changes in serum ACE2 and Ang-(1-7) concentrations after OT in AHF patients with reduced ejection fraction (EF).

**Methods:** ACE2 and Ang-(1-7) concentrations were measured in 68 AHF patients with reduced EF immediately after admission and 1 and 3 months after OT. These parameters were compared with the healthy individuals at three time points.

**Results:** In the acute phase, Ang-(1-7) and ACE2 concentrations was statistically significantly lower and higher in AHF patients than the healthy individuals (2.40 ± 1.11 vs. 3.1 ± 1.1 ng/ml, *P*<0.005 and 7.45 ± 3.13 vs. 4.84 ± 2.25 ng/ml, *P*<0.005), respectively. At 1 month after OT, Ang-(1-7) concentration remained lower in AHF patients than the healthy individuals (2.37 ± 1.63 vs. 3.1 ± 1.1 ng/ml, *P*<0.05); however, there was no statistically significant difference in ACE2 concentration between AHF patients and the healthy individuals. At 3 months after OT, there were no statistically significant differences in Ang-(1-7) and ACE2 concentrations between AHF patients and the healthy individuals.

**Conclusion:** ACE2 concentration was equivalent between AHF patients and the healthy individuals at 1 and 3 months after OT, and Ang-(1-7) concentration was equivalent at 3 months after OT.

## Introduction

Studies have shown that the renin–angiotensin system (RAS) is associated with worsening of hypertension and various types of organopathies via the angiotensin-converting enzyme (ACE)/angiotensin (Ang) II (Ang II)/Ang II type 1 receptor axis [1,2]. Long-term activation of the neurohormonal response, especially of the sympathetic nervous system and the renin–angiotensin–aldosterone system, is a major molecular hallmark of adverse LV remodeling. In fact, the levels of both plasma catecholamines and aldosterone are important predictors of cardiovascular mortality in post-MI patients [3,4].

**Figure 1:**
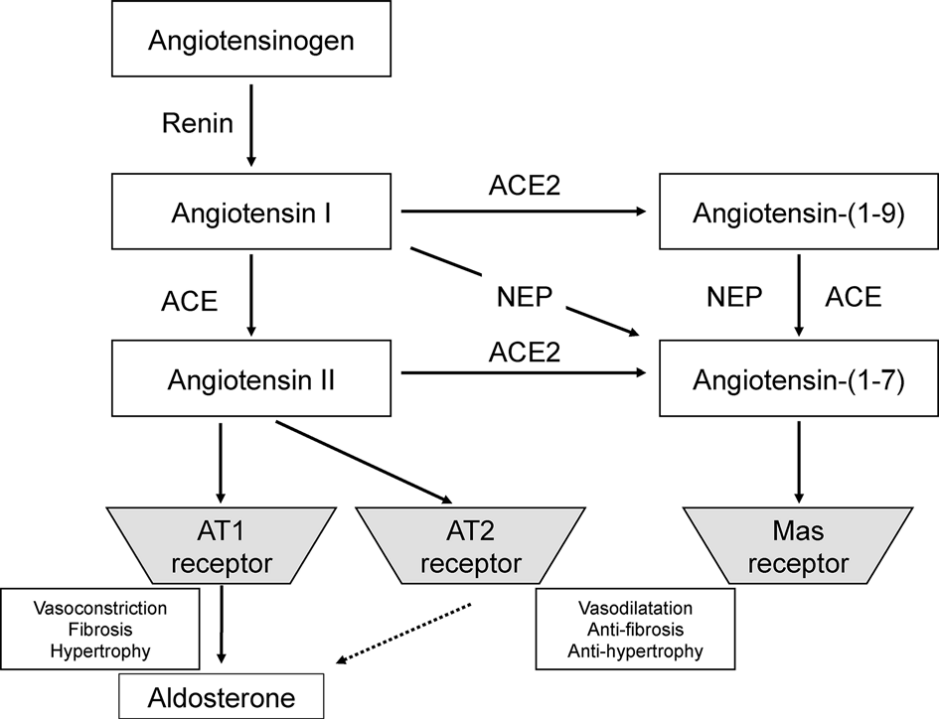
The molecular pathways of conventional ACE/Ang II/Aldosterone and the novel ACE2/Ang-(1-7) ACE2 was identified as a major Ang-(1-7)-forming enzyme. Ang I serves as a substrate for both ACE and ACE2. Ang II is known to have vasoconstrictor and fibrotic and hypertrophic effects *in vivo*. Both ACE and ACE2 are involved in the production of Ang-(1-7), which binds the Mas receptor and induces vasodilation, anti-fibrosis and anti-hypetrophy. Abbreviations: ACE, angiotensin converting enzyme; AT, angiotensin Ⅱ type; NEP, neutral endopeptidase.

Recent studies have established the existence of a new cascade in the RAS called the ACE2/Ang-(1-7)/Mas receptor axis [5,6]. Ang-(1-7) is a degradation product of Ang I and Ang II associated with ACE2. Studies in mice have reported that Ang-(1-7) stimulates the Mas receptor and induces hypotensive effects and organ-protective effects primarily through vasodilation, natriuresis, regulation of myocardial hypertrophy and anti-inflammation [7–12]. Therefore, Ang-(1-7) has antagonistic influences on the effects induced by the Ang II type 1 receptor (Figure 1).

We previously reported that acute heart failure (AHF) patients requiring hospitalization had higher serum ACE2 and lower Ang-(1-7) concentrations in the acute phase when compared with the concentrations in healthy volunteers [13]. Furthermore, it is very interesting to evaluate the variability of serum ACE2 and Ang-(1-7) concentrations in the process of compensating for heart failure. However, no study has reported serial changes in serum ACE2 and Ang-(1-7) concentrations after optimal therapy (OT) in these patients. Because of serum ACE2 and Ang-(1-7) concentrations may be biomarkers that reflect the pathogenesis of heart failure, significance of measuring ACE2 and Ang-(1-7) concentrations in patients with compensated heart failure, is very important. The present study aimed to investigate the serial changes in the serum ACE2 and Ang-(1-7) concentrations after OT in AHF patients requiring inpatient care.

Also, due to the recent worldwide pandemic of COVID-19, investigation regarding role of ACE2 has been in the limelight.

## Materials and methods

### Study design

Among patients who were urgently admitted to our hospital because of AHF with reduced left ventricular ejection fraction (LVEF) between November 2012 and September 2015, we enrolled 68 patients who consented to participate in the present study and were followed for at least 3 months. AHF was diagnosed according to the Framingham criteria [14]. Patients received optimal drug therapy according to previous guidelines [15], during hospitalization and after discharge as outpatients.

The exclusion criteria were as follows: (1) LVEF over 40%; (2) age < 20 years; (3) need for mechanical support; (4) renal failure (serum creatinine level > 3.0 mg/dl); (5) pregnancy, possible pregnancy, nursing and desire to become pregnant during the study period; (6) judgement as inappropriate for the present study by responsible doctors.

The patients were treated in accordance with the Declaration of Helsinki after obtaining informed consent. The study protocol was approved by the Ethics Committee of Toho University Omori Hospital, Japan (application number 25-229).

### Primary endpoints

The reference values of serum ACE 2 and Ang-(1-7) concentrations have not been established so far. Therefore, the value of the concentrations in healthy volunteers [13] was defined as the healthy individuals. The primary end points of the present study were the serum Ang-(1-7) and ACE2 concentrations at 1 and 3 months later after OT in the patients were compared with the healthy individuals.

### Blood sampling

Upper limb venous blood samples were percutaneously collected while AHF patients maintained the sitting posture for more than 30 min after they became capable of sitting in the acute phase. Similarly, upper limb venous blood samples were percutaneously collected 1 and 3 months after OT for heart failure while the patients maintained the sitting posture for more than 30 min.

The serum ACE2 concentration, Ang-(1-7) concentration plasma aldosterone concentration and plasma brain natriuretic peptide (BNP) concentration were measured. The reagents and detailed method for each test are described in past report [13]. Additionally, serum ACE2 and Ang-(1-7) concentrations at three time points were compared with the healthy individuals, which have been reported previously [13].

After collection, all blood samples were immediately centrifuged at 3000 rpm for 10 min in our laboratory. Dispensed specimens were frozen in dry ice. On the day of blood collection, specimens were brought to LSI Medience Corporation and were preserved in a deep freezer until measurement. ACE2 was measured without specimen dilution, and Ang-(1-7) was measured after diluting the specimen five times. With regard to cross-reactivity, we did not perform enzyme treatment, but all blood samples were immediately frozen. Serum ACE2 and Ang-(1-7) concentrations were measured using ELISA kits (Peninsula Laboratories, LLC, San Carlos, CA), according to the manufacturer’s instructions, except for solid phase extraction (SPE). We did not extract Ang-(1-7) from serum samples using SPE. After blood separation, all experiments were performed at LSI Medience Corporation using the following kits: ACE2 (Cat. No. AG-45A-0022EK-KI01; Adipogen, San Diego, CA) and Ang-(1-7) (Cat. No. S-1330 Angiotensin I/II (1-7); Peninsula Laboratories, LLC).

### Echocardiographic measurements

Transthoracic echocardiography was performed using echocardiograph on admission (in resting conditions). Two dimensional imaging was performed in standard apical views. The LVEF was measured by Simpson method. Two experienced cardiologists unaware of the biochemical data performed the echocardiographic measurements.

### Statistical analysis

All data are expressed as mean ± standard deviation (SD). Serial changes in the plasma aldosterone concentration and BNP concentration were analyzed using one-way analysis of variance (ANOVA). For the serum ACE2 concentration and Ang-(1-7) concentration, values at baseline, 1 and 3 months later were analyzed using the unpaired Student’s *t* test and were compared with the healthy individuals, which were reported previously [13]. The unpaired Student’s *t* test and ANOVA were performed using SPSS software (ver. 20.0; IBM Corp., Armonk, NY). Statistical significance was set at a *P*-value <0.05.

## Results

**Figure 2:**
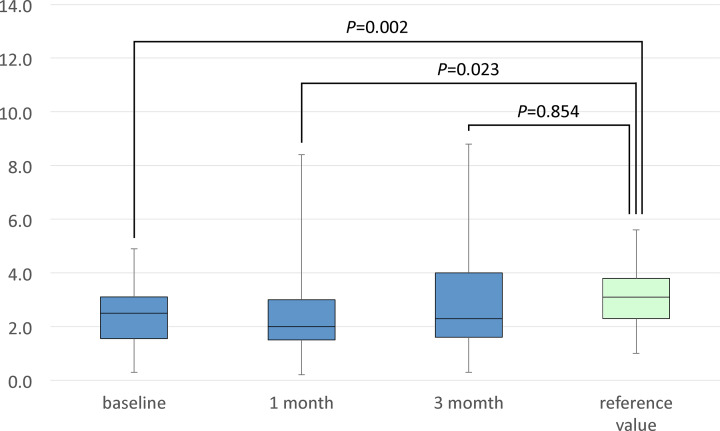
Comparison of the serum Ang-(1-7) concentration between patients and the reference value

**Figure 3:**
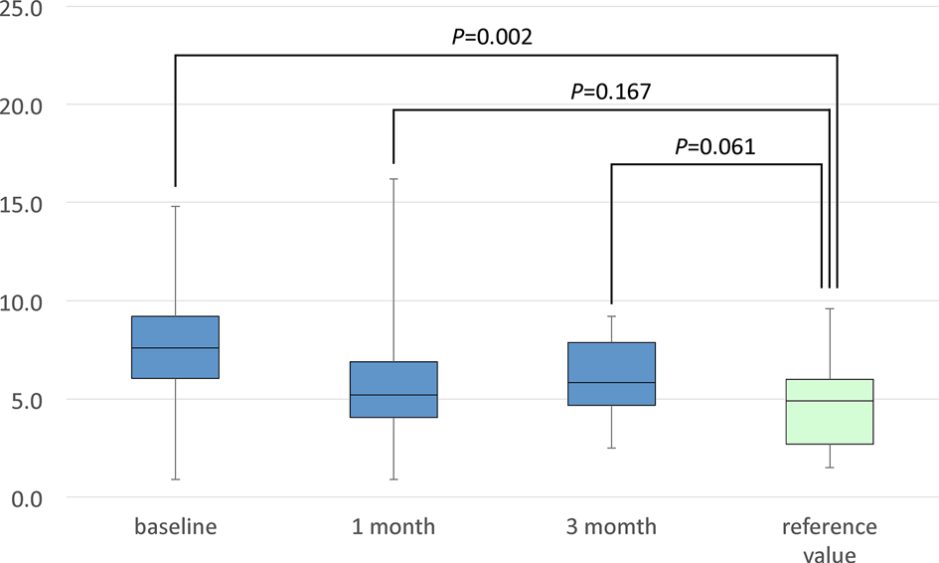
Comparison of the serum ACE2 concentration between patients and the reference value

The clinical characteristics of the AHF patients are presented in Table 1. The contents of optimal medical therapy are described in Table 2. The clinical characteristics of healthy volunteers are shown in Table 3. The plasma aldosterone concentrations at 1 and 3 months after OT were statistically significantly elevated when compared with the concentration at baseline (120 ± 57 and 120 ± 58 vs. 75 ± 41 pg/ml, respectively, *P*<0.001). The plasma BNP concentrations at 1 and 3 months after OT were statistically significantly decreased when compared with the concentration at baseline (185 ± 156 and 143 ± 139 vs. 682 ± 561 pg/ml, respectively, *P*<0.001; Table 4). In the acute phase, the serum Ang-(1-7) concentration was statistically significantly lower and the serum ACE2 concentration was statistically significantly higher in heart failure patients than the healthy individuals (2.40 ± 1.11 vs. 3.10 ± 1.10 ng/ml, *P*<0.005 and 7.45 ± 3.13 vs. 4.84 ± 2.25 ng/ml, *P*<0.005, respectively; Figures 2 and 3). At 1 month after OT, the serum Ang-(1-7) concentration remained lower in heart failure patients than the healthy individuals (2.37 ± 1.63 vs. 3.10 ± 1.10 ng/ml, *P*<0.05; Figure 2). However, there was no statistically significant difference in the serum ACE2 concentration between heart failure patients and the healthy individuals (6.11 ± 3.36 vs. 4.84 ± 2.25 ng/ml, *P*=0.167; Figure 3). At 3 months after OT, there were no statistically significant differences in the serum Ang-(1-7) and ACE2 concentrations between heart failure patients and the healthy individuals (3.03 ± 2.07 vs. 3.10 ± 1.10 ng/ml, *P*=0.854 and 6.10 ± 2.04 vs. 4.84 ± 2.25 ng/ml, *P*=0.061, respectively; Figures 2 and 3).

**Table 1:** AHF patient clinical characteristics

	Patients with AHF
Number	68
Age (years)	64 ± 14
Sex (male/female)	51/17
Height (cm)	164 ± 9
Body weight (kg)	68 ± 20
Body mass index (kg/m^2^)	25 ± 6
Systolic blood pressure (mmHg)	119 ± 24
Diastolic blood pressure (mmHg)	69 ± 16
LVEF (%)	34 ± 5
Cause of heart failure	
Ischemic	35 (51%)
Non-ischemic	33 (49%)
New York Heart Association Class	
III	30 (44%)
IV	38 (56%)
Medication before hospitalization	
ACE-i/ARB	19 (28%)
β-blockers	14 (21%)
Loop diuretics	6 (9%)
Mineralocorticoid receptor antagonists	5 (7%)
Tolvaptan	1 (1%)
Calcium channel blockers	13 (19%)
Nitrates	4 (6%)
Carperitide	0 (0%)
Inotropic agents	2 (3%)
Diabetes mellitus	24 (35%)
Lipid disorder	26 (38%)
Smoking	29 (43%)
Obesity (body mass index > 25 (kg/m^2^))	23 (33%)
BUN (mg/dl)	19 ± 13
Cr (mg/dl)	1.10 ± 0.80
eGFR (ml/min/1.73 m^2^)	64.1 ± 24.5
HbA1c (NGSP) (%)	6.1 ± 1.0
TC (mg/dl)	170 ± 42
LDL-C (mg/dl)	106 ± 34
HDL-C (mg/dl)	49 ± 17
LDL-C/HDL-C	2.36 ± 0.95
Triglyceride (mg/dl)	97 ± 63

Data are given as mean ± SD or *n* (%). Abbreviations: ACE-i, angiotensin converting enzyme inhibitor; ARB, angiotensin receptor blocker; BUN, blood urea nitrogen; Cr, creatinine; eGFR, estimated glomerular filtration; HbA1c, hemoglobin A1c; HDL-C, high-density lipoprotein cholesterol; LDL-C, low-density lipoprotein cholesterol; NGSP, National Glycohemoglobin Standardization Program; TC, total cholesterol.

**Table 2:** Optimal medical therapy (continuous or discontinuous)

Drug	
ACE-i/ARB	56 (82%)
β blockers	61 (90%)
Loop diuretics	38 (56%)
Mineralocorticoid receptor antagonists	18 (26%)
Tolvaptan	6 (9%)
Calcium channel blockers	16 (24%)
Nitrates	36 (53%)
Carperitide	31 (46%)
Inotropic agents	14 (21%)

Data are given as *n* (%). Abbreviations: ACE-i, angiotensin converting enzyme inhibitor; ARB, angiotensin receptor blocker.

**Table 3:** Healthy volunteers clinical characteristics

	Healthy volunteers
Number	38
Age (years)	47 ± 12
Sex (male/female)	35/3
Height (cm)	171 ± 6
Body weight (kg)	67 ± 12
Body mass index (kg/m^2^)	23 ± 3
Systolic blood pressure (mmHg)	124 ± 74
Diastolic blood pressure (mmHg)	78 ± 10
Concomitant drug	
Calcium channel blocker	1 (3%)
β blocker	1 (3%)
Diuretics	0
Diabetes mellitus	0
Lipid disorder	3 (8%)
Smoking	4 (11%)
Obesity (body mass index > 25 (kg/m^2^))	5 (13%)
BUN (mg/dl)	13 ± 3
Cr (mg/dl)	0.80 ± 0.11
eGFR (ml/min/1.73 m^2^)	80.9 ± 13.3
HbA1c (NGSP) (%)	5.4 ± 0.4
TC (mg/dl)	201 ± 32
LDL-C (mg/dl)	120 ± 32
HDL-C (mg/dl)	66 ± 21
LDL-C/HDL-C	2.03 ± 0.87
Triglyceride (mg/dl)	99 ± 50

Data are given as mean ± SD or *n* (%). Abbreviations: BUN, blood urea nitrogen; Cr, creatinine; eGFR, estimated glomerular filtration; HbA1c, hemoglobin A1c; HDL-C, high-density lipoprotein cholesterol; LDL-C, low-density lipoprotein cholesterol; NGSP, National Glycohemoglobin Standardization Program; TC, total cholesterol.

**Table 4:** Serial changes in plasma aldosterone concentration and plasma BNP concentration

	Baseline	1 month	3 months	*P*-value
Plasma aldosterone concentration (pg/ml)	75 ± 41	120 ± 57*	120 ± 58*	<0.001
Plasma BNP concentration (pg/ml)	682 ± 561	185 ± 156*	143 ± 139*	<0.001

Data are given as mean ± SD or *n* (%).

* <0.001 (vs. baseline).

**Figure 4:**
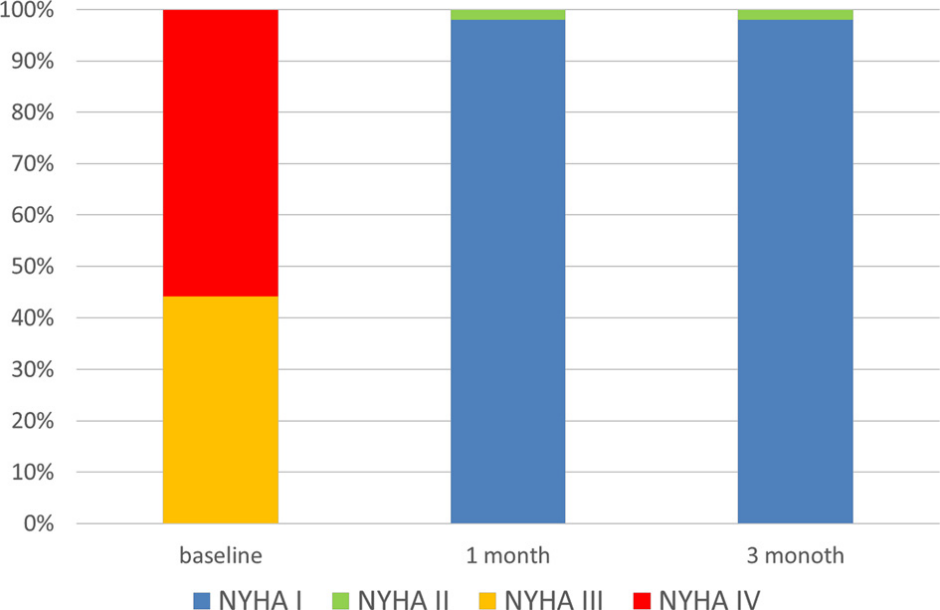
Serial changes in the NYHA classification

Serial changes in the New York Heart Association (NYHA) classification are shown in Figure 4.

## Discussion

In the present study, all cases of decompensated heart failure with reduced EF were treated with OT following the appropriate guidelines [15], and they were all compensated along with a statistically significant decrease in the plasma BNP concentration.

We previously reported that acute decompensated heart failure patients had a statistically significantly higher serum ACE2 concentration and a statistically significantly lower serum Ang-(1-7) concentration when compared with the concentrations in healthy volunteers [13]. At 1 month after OT, there was no significant difference in the serum ACE2 concentration between heart failure patients and the healthy individuals. At 3 months after OT, the statistically significant difference in the serum Ang-(1-7) concentration between heart failure patients and the healthy individuals disappeared.

Although the present study did not elucidate the mechanisms of the changes in ACE2 and Ang-(1-7) concentrations, it showed that the serum ACE2 concentration declined and the Ang-(1-7) concentration increased by OT in compensated heart failure cases, and as a result, both concentrations were equivalent to the healthy individuals. To our knowledge, this is the first study to report such findings.

It is well known that classical cascade in the RAS called the ACE/Ang II/Ang II type 1 receptor axis is a facilitating factor that promotes hypertension and damage to a variety of organs [1,2]. On the other hand, the existence of Ang-(1-7) was first reported in 1988 [16]. Crackower et al. reported in 2002 that ACE2 is involved in the development of heart failure in animal study [7]. Although the Ang-(1-7) receptor has long been unknown, it was confirmed by Santos et al. in 2003 that the Mas receptor is the Ang-(1-7) receptor [8]. Based on these researches, it was shown that the ACE2/Ang-(1-7)/Mas receptor system maintains the mutual relationship by the action of Ang II mediated by Ang II type 1 receptor and has an action to antagonize the ACE/Ang II/Ang II type 1 receptor system [5,6]. Ang-(1-7) is produced from Ang I and Ang II catalyzed by ACE2. Ang II exerts actions such as vasoconstriction, inducing oxidative stress, cell hypertrophy, and fibrosis via Ang II type 1 receptor. Ang-(1–7) is known to exert anti-hypertensive and organ-protective effects via Mas receptor through vasodilation, reduction in oxidative stress, suppression of cell hypertrophy, anti-fibrotic action, natriuresis and anti-inflammation, it is known that the relationship between Mas receptor and Ang II type 1 receptors shows an antagonistic action like a seesaw mechanism [7–12].

A previous animal experiment showed that the ACE2 concentration increases if Ang II increases [17]. Ang-(1-7) has been shown to have a cardiovascular protective effect. Among the few studies that reported on the hypertensive or hypotensive effects induced by a decrease in the ACE2 concentration, Yamamoto et al. reported that ACE2 kinetics does not have a direct effect on blood pressure [18]. The results of this study suggest that Ang-(1-7), which has a cardiovascular protective effect, is insufficient in the decompensated heart failure stage, and its concentration increases with OT. Additionally, an increased ACE2 concentration is thought to decline to an optimal level, which is associated with sufficient Ang-(1-7).

Furthermore, a previous study reported that the Ang-(1-7) concentration increases according to ACE2 activity in human heart failure [19]; however, the Ang-(1-7) concentration increased during the compensation process of decompensated heart failure in our study. The findings indicate that Ang-(1-7) is relatively insufficient in decompensated heart failure in the acute phase and the concentration increases along with compensation by OT.

These findings are very advanced results that favorably add to the further clarification of the mechanisms of the efficacy of the Ang II type 1 receptor blocker/neutral endopeptidase inhibitor combination [20], which has recently been approved for heart failure in Europe and the U.S.A.

In the present study, 82% of patients were treated with ACE-inhibitor (ACEi) or angiotensin receptor blocker (ARB). Therefore, it was expected that the plasma aldosterone concentration after discharge was lower than that at baseline. However, contrary to that expectation, plasma aldosterone concentrations increased after discharge, and the values at 1 and 3 months after OT were similar. We observed a gradual increase in the plasma aldosterone concentration along with compensation of heart failure in the present study. A previous study by Takeda et al. [21] reported that the circulating aldosterone concentration was inversely proportional to the myocardial tissue aldosterone concentration in an animal experiment. It can be assumed that a decrease in the myocardial tissue aldosterone concentration caused by compensation of heart failure was indirectly observed. In the present study, 26% of patients were treated with mineralocorticoid receptor antagonists (MRAs). It can be explained that MRAs administration increased plasma aldosterone concentration in these patients. The mechanism of elevated plasma aldosterone levels in patients who did not receive MRA is difficult to explain. According to a report by Mizuno et al., aldosterone acts as a cardioprotective effect for a short period [22]. Therefore, it is considered that the concentration increases in the early phase of decompensated AHF, and the transition may be different for each patient due to the damage of myocardium when shifting to the chronic phase. However, there have been almost no reports to date that measured plasma aldosterone concentrations in patients with heart failure from the acute phase to the chronic phase. In addition, the reason for the increase in plasma aldosterone concentration after discharge in present study may be the effect of the aldosterone breakthrough phenomenon [23–25]. Specifically, after the RAS cascade is inhibited by ACE-i or ARB, the concentration of aldosterone at the most downstream of RAS is temporarily reduced. After that, the compensatory mechanism (details unknown) raises the aldosterone concentration again. It is considered that the aldosterone concentration changes similarly even when MRA is administered, and the aldosterone concentration increases because it does not bind to the mineralocorticoid receptor. Recently, it has been known that BNP has an action of suppressing the secretion of aldosterone from the adrenal gland [26]. The mechanism by which the suppression of aldosterone secretion is relieved by the reduction in BNP by compensating heart failure may also influence the increase in aldosterone levels. Moreover, there is also a report that administration of ACE-i or ARB increases the secretion of aldosterone via Ang II type 2 receptor [27], and our results may support this report. However, this cannot be affirmed only by the data of present study.

Further evidence from studies, including those involving animal experiments, is necessary as causal relationships between the kinetics of biomarkers in circulating blood and the concentrations of various biomarkers in tissue are unclear.

Finally, with recent pandemic of COVID-19, some concerns about the use of ACE-i and ARB have emerged in COVID-19 patients. ACE2 is the molecule used by severe acute respiratory syndrome coronavirus 2 (SARS-CoV-2) responsible for COVID-19 pandemic to enter the cells [28–32]. Therefore, optimal medical treatment in heart failure, by reducing ACE2, will decrease the entrance of SARS-CoV-2 in the cells and thus mitigate the severity of the disease [33]. Contrary to initial reports by mass media that ACE-i/ARB might be deleterious in Covid-19, use of either may be associated with a lower risk of in-hospital deaths than nonuse.

## Limitations of the present study

The present study has some limitations. First, the study included limited number of diseased. Second, with regard to ACE2, the evaluation of activity rather than concentration might have allowed better and more direct understanding of the ACE2 effect; however, we could not obtain an ACE2 activity assay kit for the present study. We consider that the ACE2 concentration should reflect ACE2 activity. Moreover, although ACE2 activity was not measured, we believe that measurement of the Ang-(1-7) concentration, which is a product of ACE2, supports the significance of the present study. Third, in the present study, we could not evaluate the effects of the drugs used for hospitalization treatment. Therefore, it is impossible to mention the effects on various parameters of drugs. Fourth, the patients were not treated with the modern SGLT2 inhibitors that have demonstrated to improve prognosis [34]. This is important because SGLT2 inhibitors mitigates this neurohormonal activation [35].

## Conclusions

The serum ACE2 concentration was equivalent between AHF patients and the healthy individuals at 1 and 3 months after OT. Furthermore, the serum Ang-(1-7) concentration was equivalent between AHF patients and the healthy individuals at 3 months after OT. In other words, it was observed that the increased serum ACE2 concentration gradually decreased to normal value, and the decreased serum Ang-(1-7) concentration gradually increased to normal value, in patients with acute decompensated heart failure with reduced LVEF, who were compensated after optimal medical therapy based on guidelines.

## Abbreviations

ACE: angiotensin-converting enzyme
ACE-i: angiotensin-converting enzyme inhibitor
AHF: acute heart failure
Ang: angiotensin
Ang-(1-7): angiotensin-(1-7)
Ang II: angiotensin II
ANOVA: analysis of variance
ARB: angiotensin II receptor blocker
BNP: brain natriuretic peptide
EF: ejection fraction
LVEF: left ventricular ejection fraction
MI: myocardial infarction
MRA: mineralocorticoid receptor antagonist
OT: optimal therapy
RAS: renin–angiotensin system
SARS-CoV-2: severe acute respiratory syndrome coronavirus 2
SGLT2: sodium glucose cotransporter 2
SPE: solid phase extraction
